# In-vitro antimalarial activity of methanolic leaf- and stem-derived extracts from four *Annonaceae* plants

**DOI:** 10.1186/s13104-023-06664-w

**Published:** 2023-12-22

**Authors:** Pathrapol Lithanatudom, Kriangkrai Chawansuntati, Chalermpong Saenjum, Tanawat Chaowasku, Kritsadee Rattanathammethee, Boonsong Wungsintaweekul, Maslin Osathanunkul, Jiraprapa Wipasa

**Affiliations:** 1 Department of Biology, Faculty of Science, Chiang Mai University, Chiang Mai, 50200 Thailand; 2 Research Center in Bioresources for Agriculture, Industry and Medicine, Faculty of Science, Chiang Mai University, Chiang Mai, 50200 Thailand; 3 Research Institute for Health Sciences, Chiang Mai University, Chiang Mai, 50200 Thailand; 4 Department of Pharmaceutical Science, Faculty of Pharmacy, Chiang Mai University, Chiang Mai, 50200 Thailand; 5 School of Pharmacy, Walailak University, Nakhon Si Thammarat, 80160 Thailand

**Keywords:** *Annonaceae*, Malaria, *Plasmodium falciparum*, *Artabotrys burmanicus*, *Marsypopetalum modestum*

## Abstract

**Objective:**

Plants in the *Annonaceae* family are known for having abundant biologically active secondary metabolites. They have been used in alternative drugs for various diseases in several countries, for instance, the bark of *Cananga odorata (Lam.)* Hook and Thomson is used for Ophthalmic inflammation and wound healing in Malaysia. Extracts from the leaves and stems of four *Annonaceae* plants, namely *Uvaria longipes* (Craib) L.L.Zhou, Y.C.F.Su & R.M.K.Saunders, *Dasymaschalon* sp., *Artabotrys burmanicus* A.DC, and *Marsypopetalum modestum* (Pierre) B.Xue & R.M.K.Saunders were investigated for growth inhibitory activity against blood-stage *Plasmodium falciparum* growth in vitro and for non-specific cytotoxicity against normal peripheral blood mononuclear cells (PBMCs). Antimalarial activity was assessed by invasion inhibition assay and the percentage of infected red blood cells on blood smears were determined. Cytotoxicity was tested by culturing PBMCs with the extracts, and viabilities were determined by Annexin V/propidium iodide staining.

**Results:**

*A. burmanicus* stem extract and *M. modestum* leaf extract were capable of inhibiting growth of *P. falciparum* when used at 200 µg/mL compared to chloroquine. The extracts at effective concentrations, did not affect the viability of PBMCs. These results support further need for characterization of active compounds from specific *Annonaceae* plants in order to exploit their components for potential malaria treatment.

## Introduction

For centuries, plant-derived active compounds have been explored extensively for medicinal use. There are approximately 374,000 plant species characterized so far [1]. The family *Annonaceae*, comprising 112 genera and 2,440 species [2], provides numerous bioactive compounds for ethnomedical use including therapeutic drugs to treat several diseases, including cancer, inflammatory diseases, and malaria, etc. [3]. It has been reported as a rich source for alkaloids, terpenoids, acetogenins and other bioactive compounds [4].

The most effective antimalarial drug available at present was first isolated from a plant species, namely *Artemisia annua* [5]. This natural active compound was termed artemisinin, and its derivatives (artesunate, artemether, arteether, and dihydroartemisinin) have been used in the treatment of *Plasmodium falciparum* malaria. The quinoline alkaloid quinine has also been used as the standard treatment for malaria for decades and this compound was isolated from the bark of the *Cinchona* plant family [6]. Monotherapy often results in drug resistance, thus a first-line treatment with artemisinin-based combination therapies (ACTs) have been recommended by the World Health Organization [7]. Although these drugs have proven highly effective and successfully reduced malaria-associated mortality and morbidity, drug-resistant malaria has begun to emerge [8]. Therefore, the development of a novel antimalarial drug is required to combat malaria. One systematic review showed that 63 species from 27 genera of the *Annonaceae* family have been tested for antimalarial activity [9]. Ethanolic extracts from *Polyalthia debilis* and *Xylopia aromatica* were reported as having inhibitory activity against *P. falciparum* growth in vitro with median inhibition concentration, IC_50_ < 1.5 µg/mL [9]. However, given that the *Annonaceae* family consists of 2,440 species, numerous species from this family remain to be explored.

Several species of the *Annonaceae* family have been used in traditional medicine in Southeast Asian countries, including Thailand. The stem and root of *Marsypopetalum modestum* have been used as anti-tuberculosis treatment by ethnic groups in Laos [10]. *Uvaria longipes* stems have been known to have aphrodisiac properties by ethnic groups in eastern and northeastern Thailand (personal interviews). These ethnic groups also use the stems of *Dasymaschalon* sp. and *D. lomentaceum* to relieve muscular pain [11]. The stem bark of *Artabotrys burmanicus* has been used as a heart tonic by ethnic groups in southwestern Thailand (personal interviews). In this study, we aimed at screening leaf- and stem-derived crude extracts from four *Annonaceae* plants, including *Uvaria longipes, Dasymaschalon* sp. (a new species to be segregated from *D. lomentaceum*; manuscript in preparation), *Artabotrys burmanicus* and *Marsypopetalum modestum* for antimalarial activity. Cytotoxicity of the extracts was also assessed to determine potential undesirable side effects.

## Materials and methods

### Reagents and chemicals

Rosmarinic acid, luteolin and apigenin were obtained from Sigma-Aldrich (St. Louis, MO, USA). Caffeic acid, rutin, quercetin and kaempferol were purchased from Tokyo Chemical Industry Co., Ltd. (Tokyo, Japan).

### Plant materials and extraction

Four species of Annonaceous plants were collected once for each species from a private garden at coordinates 13.919300, 99.952555. as previously described [12, 13]. Voucher specimens were identified by TC and deposited at CMUB herbarium, Department of Biology, Faculty of Science, Chiang Mai University. The leaves and stem of the samples were cleaned and dried at 50 °C. Each sample was milled into a powder, which was subsequently extracted with refluxing methanol (1,000 g/4,000 mL). The combined methanol fractions were filtered with filter paper, before all volatiles were removed from the filtrate under reduced pressure in a rotary evaporator to yield the crude methanolic extract. The extracts were dissolved in dimethyl sulfoxide (DMSO) (Gibco-BRL), and were diluted with 10% fetal calf serum/RPMI 1640 (Gib-co-BRL) media to desired concentrations prior to assays.

### Chromatographic analysis for phenolic and flavonoid compounds

The reversed-phase HPLC, Agilent 1200 equipped with the multi-wavelength detector was used in the analysis of phenolic and flavonoid compounds namely gallic acid, caffeic acid, rutin, rosmarinic acid, luteolin, quercetin, and kaempferol, as previously described [14]. The analysis was carried out using a SymmetryShield RP18 column (4.6 mm × 250 mm, 5 μm particle diameters, Waters Co., Ltd.). The mobile phase consisted of 30% acetonitrile in 0.1% acetic acid and de-ionized water using isocratic elution at the flow rate of 1.0 mL/min. The injection volume of each reference standard and samples in the solvent (10 mg/mL) was 10 µL. The content peaks were detected using a UV detector at 220 nm (gallic acid and caffeic acid) and at 325 nm (rutin, rosmarinic acid, luteolin, quercetin, apigenin, and kaemferol). The calibration curve of each compound was used. The linearity range of gallic acid and caffeic acid was 6.25–100.0 µg/mL. Additionally, the linearity range of rutin, rosmarinic acid, luteolin, quercetin, apigenin, and kaempferol was 2.50–50.0 µg/mL. The amounts of each detected compound in the samples were calculated and expressed as mg/g extract.

### Chromatographic analysis for acetogenin compounds in stem extract

The stem extract was analyzed for acetogenin compounds namely, annoglaxin, bullatacin, squamocin, asiminecin, and murisolin following the HPLC condition reported by Yang et al. [15] with slight modifications. The analysis was carried out using a Symmetry RP18 column (4.6 mm × 250 mm, 5 μm particle diameters, Waters Co., Ltd.). The mobile phase consisted of 75% methanol in de-ionized water with using isocratic elution at the flow rate of 1.0 mL/min. The injection volume of each reference standard and samples (10 mg/mL) was 10 µL and the column temperature was set at 30 °C. The content peaks were detected using a UV detector at 220 nm and the amounts of each detected compound in the samples were calculated and expressed as mg/g extract.

### *P. falciparum* invasion assay

The *P. falciparum* 3D7 strain was maintained in a continuous culture under standard conditions [16]. Mature schizonts were obtained by gradient centrifugation over 60% Percoll (GE Healthcare Life Science, Buckinghamshire, England). Isolated schizont-infected red blood cells (RBCs) at 1 × 10^7^ cells were added into 1 × 10^9^ RBCs (1% parasitemia) and cultured in the presence of methanolic extracts at the concentrations of 200 µg/mL, 100 µg/mL, 50 µg/mL and 25 µg/mL. Parasites cultured in the presence of 2 µg/mL chloroquine, 1% DMSO or medium alone were used as controls. After 96 h, blood smears were made and randomly assigned numbers on each slide. The smears were blindly reviewed by two microscopists and the average percentage of infected RBCs were determined. The assays were repeated three times.

### Cytotoxicity assay

Peripheral blood mononuclear cells (PBMCs) were isolated from five healthy donors by gradient centrifugation over Ficoll-Hypaque (Biochrom, Germany). The cells were cultured for 96 h in the presence of the extracts at the concentrations of 200 µg/mL, 100 µg/mL, 50 µg/mL and 25 µg/mL. The viability of cells was determined by Annexin (ImmunoTools GmbH, Germa-ny)/propidium iodide (PI) staining and analyzed by flow cytometry (CyAn ADP Analyzer, Beckman Coulter, USA) as described previously [12]. The assay was done in duplicate.

## Results

### *P. falciparum* growth inhibitory activity of extracts from *Annonaceae* plants

**Fig. 1:**
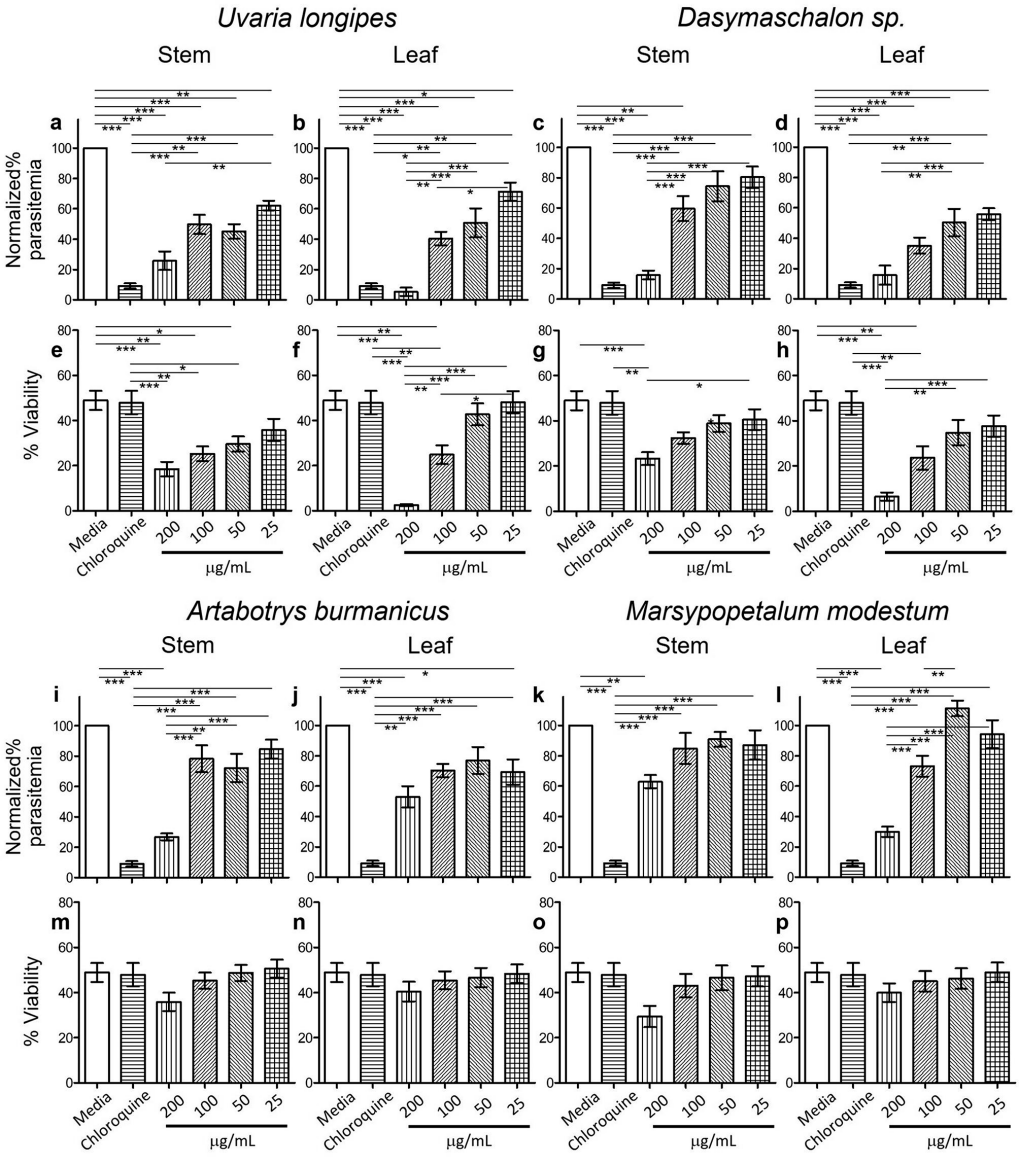
Anti-Plasmodial activity and effect on viabilities of PBMCs of extracts from the *Annonaceae*. Parasitemia of cultures in the presence of extracts from the leaves and stems of *U. longipes* (**a** and **b**), *Dasymaschalon* sp. (**c** and **d**), *A. burmanicus* (**i** and **j**), and *M. modestum* (**k** and **l**) were determined from *P. falciparum* invasion inhibition assay. The data show the mean ± SEM (N = 6). Viabilities of PBMCs cultures in the presence of extracts from the leaves and stems of *U. longipes* (**e** and **f**), *Dasymaschalon* sp. (**g** and **h**), *A. burmanicus* (**m** and **n**), and *M. modestum* (**o** and **p**) were determined by Annexin V/propidium iodide staining. The data show the mean ± SEM (N = 5)

Parasitemia from each treatment was normalized against parasitemia of medium control from the same experiment. There was no statistical difference of parasitemia between cultures in medium control and 1% DMSO (data not shown). Parasitemia of cultures in the presence of *U. longipes* stem and leaf (Fig. 1a and b) extracts exhibited statistically lower levels compared to cultures in medium alone. Parasitemia of cultures in *U. longipes* stem and leaf extracts were statistically significantly higher than that of chloroquine, except for the concentration of 200 µg/mL. The parasite growth dose-dependent inhibitory effect of *U. longipes* was more apparent in the leaf (Fig. 1b) compared to the stem (Fig. 1a). Likewise, stem and leaf extracts from *Dasymaschalon* sp. also showed parasite growth inhibitory activity in a dose-dependent manner (Fig. 1c and d). Parasitemia from cultures in the presence of *Dasymaschalon* sp. stem extracts at 200 µg/mL (Fig. 1c) and leaf extracts (Fig. 1d) at 200 and 100 µg/mL were not different from that of chloroquine.

For *A. burmanicus*, only the stem extract at 200 µg/mL (Fig. 1i) showed parasite growth inhibitory activity, which was not statistically different from chloroquine, whereas other conditions of both stem (Fig. 1i) and leaf (Fig. 1j) showed significantly higher parasitemia. Stem extract of *M. modestum* also did not show parasite inhibitory activity when compared to chloroquine (Fig. 1k). Only at a concentration of 200 µg/mL, *M. modestum* leaf extract (Fig. 1l) revealed parasitemia comparable to chloroquine.

### Evaluation of toxicity on PBMCs

Assessing cell viability is a critical step in the drug development process to determine cytotoxic effect or safety of drugs. Viable PBMCs were determined from Annexin-negative/PI-negative population. Viabilities of PBMCs cultured for 96 h in the presence of 2 µg/mL chloroquine were not different from that of medium controls (Fig. 1e − 1 h, and Fig. 1m and p). Although *U. longipes* stem and leaf extracts at 200 µg/mL (Fig. 1a and b), *Dasymaschalon* sp. stem extracts at 200 µg/mL (Fig. 1c) and leaf extracts (Fig. 1d) at 200 and 100 µg/mL showed parasite growth inhibitory activity comparable to that of chloroquine, they exerted cytotoxic effect as reflected by significant decrease in the viability of PBMCs when compared to medium control and chloroquine (Fig. 1e − 1 h). On the other hand, viabilities of PBMCs in all conditions of cultures in the presence of *A. burmanicus* and *M. modestum* stem and leaf extracts (Fig. 1m and p) were not different from those of medium controls and chloroquine. The results suggest that *A. burmanicus* stem extract and *M. modestum* leaf extract exhibited anti-malarial activity without exerting a cytotoxic effect.

### HPLC analysis of phytochemical constituents of extracts

**Fig. 2:**
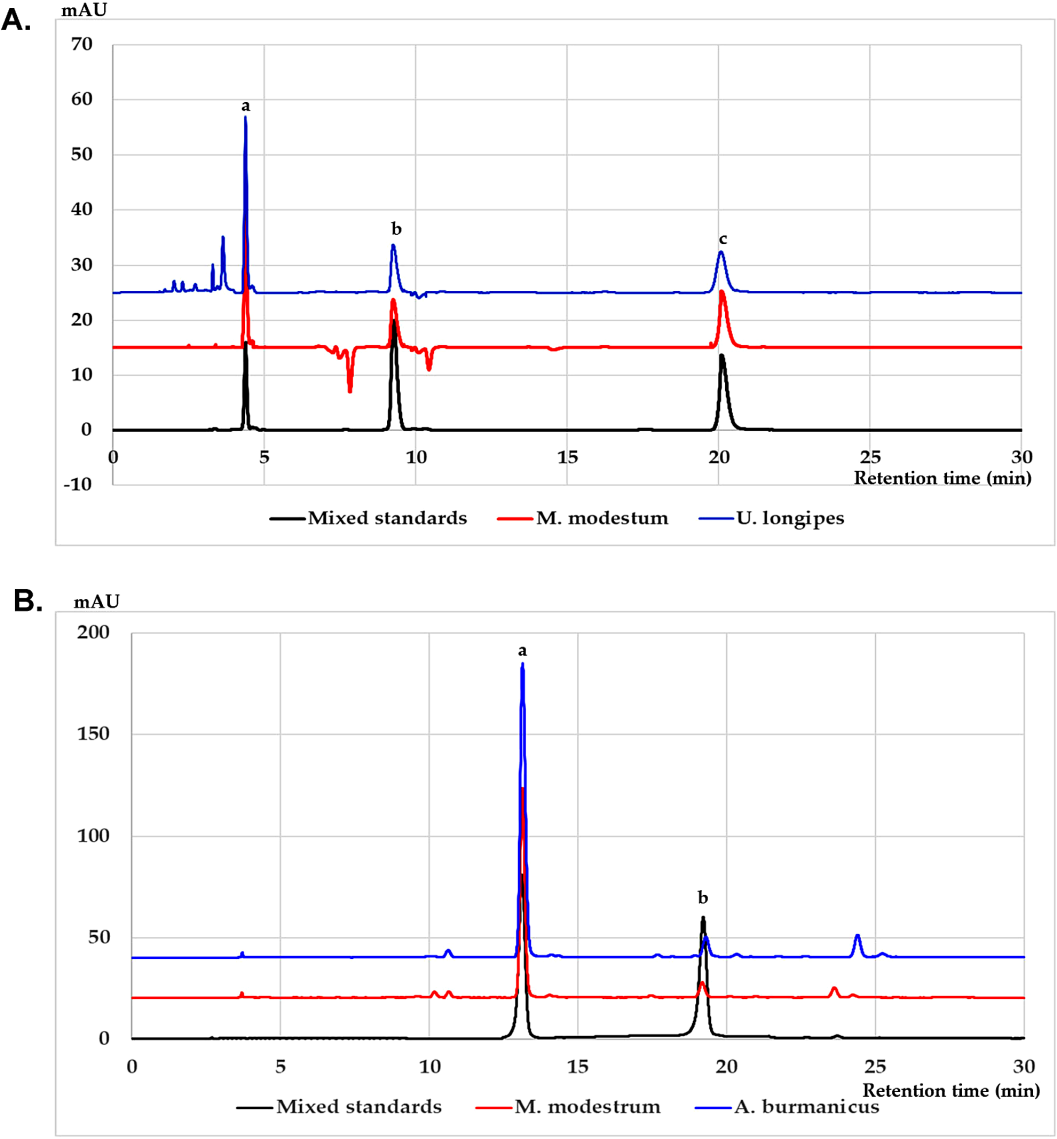
HPLC chromatograms of mixed standards, *M. modestum* and *U.longipes* leaf (**A**) and *A. burmanicus* stem (**B**) extracts. The peaks in (**A**) indicate (**a**) rutin, (**b**) rosmarinic acid, and (**c**) quercetin. The peaks in (**B**) indicate (**a**) bullatacin, and (**b**) asiminecin

We previously reported that all four leaf-derived crude extracts contained rutin and quercetin [12], in this study more reference standards were included to explore whether the extracts might contain some other active compounds. HPLC analysis confirmed that rutin and quercetin were found in leaves of all four extracts and lesser amount of rosmarinic acid was found in *U. longipes* and *M. modestrum* (Table 1). For the stem extracts, bullatacin and asiminecin were found in all four plants (Table 2) as reported previously [13]. Examples of HPLC chromatograms from *M. modestum* leave extract and *A. burmanicus* stem extract were shown in Fig. 2.

**Table 1:** The HPLC analysis of phenolic and flavonoid compounds of leaves extract^a^

Compounds	Amount (mg/g extract)			
	*U. longipes*	*A. burmanicus*	*M. modestrum*	*Dasymaschalon* sp.
Rutin Rosmarinic acid	18.05 ± 0.41 1.33 ± 0.17	6.05 ± 0.27 Nd	7.76 ± 0.23 4.62 ± 0.19	15.32 ± 0.35 Nd
Quercetin	4.19 ± 0.23	2.18 ± 0.22	9.35 ± 0.30	3.05 ± 0.17

^a^Only detected metabolites are shown

**Table 2:** The HPLC analysis of acetogenin compounds of stem extract^a^

Compounds	Amount (mg/g extract)			
	***U. longipes***	***A. burmanicus***	***M. modestrum***	***Dasymaschalon*** **sp.**
Bullatacin Asiminecin	25.22 ± 0.86 13.07 ± 0.49	34.63 ± 1.05 17.47 ± 0.68	27.32 ± 0.74 11.45 ± 0.53	21.51 ± 0.63 7.78 ± 0.72

^a^Only detected metabolites are shown

## Discussion

Although deaths due to malaria worldwide decreased more than 50% between 2000 and 2015 due to the scale up of ACTs treatment doses as well as the intervention of insecticide-treated mosquito nets in Africa, the number of clinical cases reached 228 million cases with more than 400,000 deaths in 2018 [17]. Despite tremendous efforts from multiple sectors to eradicate malaria, the World Health Organization predicts that there will still be over 10 million cases in Africa in 2050 [18]. New tools and approaches on vector control, chemotherapy and vaccines are still needed to achieve the global malaria-free goal.

Following the emergence of artemisinin-resistant *P. falciparum* malaria, investment in intensive drug discovery efforts have been made, but new classes of anti-malarial agents have yet to be developed. *Annonaceae* plants have been used for the treatment of malaria or symptoms-related to the disease in some African countries [19–21]. In this study, we investigated anti-malarial activity and cytotoxic effect of stem and leaf extracts of four *Annonaceae* plants found in Thailand. We found that stem extract of *A. burmanicus* and leaf extract of *M. modestum* were able to inhibit *P. falciparum* growth in vitro with no significant cytotoxic effect on normal PBMCs. These two extracts could be potential sources for new alternative antimalarial drugs.

High-performance liquid chromatography analysis revealed that *A. burmanicus* and *M. modestrum* leaves extract contained rutin, quercetin, and to a lesser extent [13] rosmarinic acid, whereas [12] stem extracts of these two plants contained bullatacin and asiminecin [13]. It has been reported that rutin inhibited *Plasmodium growth* both in vitro and in vivo [22]. Rutin may also be able to reduce malaria pathogenesis by modulating antioxidant and inflammatory responses [22, 23]. Quercetin alone or in-combination with other substances show efficacious for inhibiting *Plasmodium* growth [24, 25]. A molecular docking study has demonstrated that quercetim inhibits *Plasmodium* growth by inhibiting the formation of β-hematin [26]. The possible mechanism of bullatacin and asiminecin on malaria growth inhibition is not well-established. Bullatacin has been shown to inhibit cyclic AMP (cAMP) and cyclic GMP in some cell lines [27], whereas asiminecin is an inhibitor of mitochondria NADH: ubiquinone oxidoreductase [28]. Further investigations need to be carried out in order to identify the mechanisms behind antimalarial activity of bioactive constituents of *A. burmanicus* stem extract and *M. modestum* leaf extract.

While we reported other biological activities of the four *Annonaceae* used in this manuscript, we did not find anti-plasmodial activities of extracts from *Uvaria longipes* and *Dasymaschalon* sp. A previous study has shown that the leaf and fruit of *U. chamae* P. Beauv suppressed *P. berghei* replication in mice [29]. *U. acuminata* also has in vitro inhibitory effect on *P. falciparum* [30]. In the real world, *U. afzelii* is commonly used to treat symptoms of malaria [31]. Reports on anti-malarial activity of *Dasymaschalon* sp are limited. A study from Thailand reported that alkaloids and flavonoids from *D. acuminatum* were capable of inhibiting chloroquine-resistant K1 strain of *P. falciparum in vitro* [32]. The *Annonaceae* plant is, therefore, proven to be sources of potential anti-Plasmodial agents.

Although further studies are necessary to elucidate the active compounds involved and the precise pathways of the antimalarial effect, here we report that stem of *A. burmanicus* and leaf of *M. modestum* could represent potential novel routes in the drug development for malaria treatment.

## Data Availability

The datasets used and analysed during this study available from the corresponding author on reasonable request.
